# The relationship between facial negative physical self and social anxiety in college students: the role of rumination and self-compassion

**DOI:** 10.3389/fpsyg.2025.1450174

**Published:** 2025-06-03

**Authors:** Yuxian Yan, Xinyu Zhou, Jinhui Zhou, Yin Chen, Yan Zhang, Xin Zhou, Jiaming Luo

**Affiliations:** North Sichuan Medical College, Nanchong, China

**Keywords:** rumination, self-compassion, social anxiety, moderated mediation model, facial negative physical self

## Abstract

**Background:**

To investigate the association between facial negative physical self, social anxiety, rumination, and self-compassion among college students in western China.

**Methods:**

A questionnaire was used to conduct an online survey of 1, 178 students from a university in western China through convenience sampling using the Self-Compassion Scale, the Ruminative Response Scale, the Negative Physical Self Scale-facial appearance sub-scale and the Interaction Anxiousness Scale.

**Results:**

In the mediation model, the total predictive effect of facial negative physical self on social anxiety was significant (*B* = 0.46, *t* = 17.66, *p* < 0.01), and the mediating effect of facial negative physical self on social anxiety accounted for 48. 1% of the total effect; self-compassion moderated the effect of rumination on social anxiety (*B* = –0.06, *t* = 3.00, *p* < 0.01).

**Conclusion:**

Facial negative physical self affects the level of social anxiety of college students through rumination, and self-compassion regulates the effect of rumination on social anxiety. Students should be encouraged to increase their level of self-compassion or be provided with self-compassion intervention training, which can help reduce social anxiety.

## 1 Introduction

Today’s college students are in the flourishing period of adolescence, facing a new and independent interpersonal environment. On the one hand, the motivation and driving force to try to socialize are increasing; on the other hand, they do not know how to establish good interpersonal relationships, which makes social anxiety increasingly a common mental health problem of contemporary college students (Peng, 2004). According to the survey in China, 45.7% of college students have experienced social anxiety (Li et al., 2019). A meta-analysis on the global prevalence of social anxiety disorder reported prevalence rates of 8.3% in adolescents and 17% in youth populations (Salari et al., 2024). In recent years, mental health assessments among college students have revealed that a significant proportion of students experience severe social anxiety, which exerts multiple adverse effects on their well-being (Dong et al., 2024). On the one hand, mild social anxiety can lead to diminished quality of interpersonal relationships, reduced subjective well-being (Lacombe et al., 2024; Öztekin, 2024), heightened loneliness (Sun and Liu, 2018), and an increased likelihood of addictive behaviors (Huang et al., 2021). On the other hand, severe social anxiety may even contribute to clinical depression, suicidal ideation, and self-harm behaviors (Zhang and Zhou, 2018). Therefore, investigating social anxiety among college students is critical for safeguarding their mental health.

### 1.1 Theoretical framework

According to the Cognitive Behavioral Model of Social Phobia (CBMSP), the emotional responses of individuals with social anxiety are caused by non-objective and negative beliefs and perceptions (Clark and Wells, 1995). In recent years, with the widespread use of filters and P-picture software in social media, individuals’ requirements for their appearance have become higher and higher compared with the perfect image of individuals after filters, and appearance anxiety has become more common. Facial negative physical self originates from Negative Physical Self (NPS), also termed body image disturbance, which refers to an individual’s negative affective, cognitive, and behavioral responses toward their own body (Davison and McCabe, 2005). Chinese scholar Chen (2003) identified five dimensions of NPS (general, overweight, underweight, facial appearance, and short stature). As a subdimension of NPS, facial negative physical self specifically denotes negative self-cognitions about one’s facial features, encompassing the detrimental cognitive evaluations, emotional reactions, and behavioral regulations individuals develop regarding their facial appearance (Chen, 2003). Therefore, according to the CBMSP, facial negative physical self functions as a form of negative self-cognition, often play a critical role in shaping interpersonal interactions, with facial negative physical self identified as a major predictor of social anxiety (Pawijit et al., 2019). Existing research has primarily focused on the relationship between general negative physical self and social anxiety, largely overlooking the differential impacts of various NPS dimensions on social anxiety. Given that facial appearance serves as one of the most immediate and crucial factors in interpersonal interactions, this study specifically examines the influence of facial negative physical self on social anxiety and its underlying psychological mechanisms.

In exploring the relationship between facial negative physical self and social anxiety among college students, rumination—a maladaptive cognitive regulation strategy (Zhao and Zhang, 2024)—refers to an individual’s persistent focus on negative self-aspects and repetitive analysis of their causes and consequences, without actively addressing or resolving them (Watkins and Roberts, 2020). Individuals with higher levels of rumination tend to dwell excessively on negative social interactions and engage in harsh self-criticism regarding their behavioral performance, thereby amplifying anxiety in interpersonal contexts and contributing to social anxiety (Modini and Abbott, 2017). Consequently, individuals with higher levels of facial negative physical self are more susceptible to rumination due to excessive preoccupation with their negative self-perceptions, ultimately leading to social anxiety.

Previous studies have shown that self-compassion, as a positive and receptive emotion regulation strategy, has been found to have a positive impact on rumination (Bugay-Sökmez et al., 2023). In addition, previous studies on college students' social anxiety found that the core feature of social anxiety is mainly fear of negative evaluation (Swee et al., 2021), which is closely related to the concept of self-compassion. Neff (2003) proposed that self-compassion means that individuals are not hard on themselves when facing deficiencies, failures and sufferings and are full of kindness and care for themselves. Individuals with high self-compassion can make more accurate self-assessments in social situations, are less swayed by negative external evaluations (Bugay-Sökmez et al., 2023), and are better able to accept their shortcomings, thus directly countering the core characteristics of social anxiety. In addition, although the concept of self-compassion as a good way of cognitive regulation has not been proposed for a long time, numerous empirical and review studies on self-compassion have shown that self-compassion has a good effect on improving individual negative psychological states (Neff, 2023; Póka et al., 2024). For example, studies have shown that self-compassion is associated with reduced negative emotions such as anxiety, depression, and stress (Blackie and Kocovski, 2018). Therefore, we believe that when individuals cause rumination due to negative physical self-appearance, individuals with a higher level of self-compassion can alleviate social anxiety caused by rumination.Therefore, this study aims to explore the relationship between the facial negative physical self and social anxiety and discuss the roles of rumination and self-compassion in the relationship to build an internal mechanism model of the relationship between the facial negative physical self and social anxiety.

### 1.2 The relationship between facial negative physical self and social anxiety

Previous studies have demonstrated a significant association between facial negative physical self and social anxiety (IzgiçF et al., 2004; Zhu et al., 2021; Cai et al., 2024; Wan et al., 2024). Facial negative physical self encompasses negative emotional (e.g., shame, frustration), cognitive (e.g., dissatisfaction, desire for change), and behavioral (e.g., avoidance, concealment) responses toward one’s body (Chen, 2003). Consequently, when individuals are dissatisfied with their appearance, they tend to excessively focus on their looks, movements, and behaviors in social situations, perceiving that their appearance is under scrutiny by others (Menzel et al., 2010). Moreover, appearance-related insecurity may lead to overestimation of negative evaluations from others during interpersonal interactions, heightening negative emotions and resulting in tendencies toward social anxiety and social avoidance (Zhu et al., 2021). These findings are further supported by empirical research (Zhu et al., 2021; Cai et al., 2024), which indicates that individuals with facial negative physical self frequently experience anxiety and exhibit reluctance to engage in social relationships (Zhu et al., 2021). Negative self-perceptions of one’s body can also adversely affect interpersonal mental states, such as anxiety and depression (Bornioli et al., 2021). Additionally, dissatisfaction with body image has been shown to trigger fear of negative evaluation (Willemse et al., 2023), a core feature of social anxiety. Therefore, it can be hypothesized that negative physical self (including facial negative physical self) serves as a positive predictor of social anxiety and is positively correlated with it. The present study proposes Hypothesis 1: Facial negative physical self positively predicts social anxiety among college students (H1).

### 1.3 The mediating role of rumination

We propose that negative physical self of college students may directly influence social anxiety, as well as indirectly affect it through rumination. First, research on body dissatisfaction indicates that it often triggers social comparison, which significantly predicts increased rumination (Feinstein et al., 2013). This heightened rumination, in turn, exacerbates body image dissatisfaction (Etu and Gray, 2010; Rivière et al., 2018). Empirical studies have shown that individuals who hold stronger facial negative physical self tend to exhibit lower self-acceptance of their appearance and are more vulnerable to negative emotions triggered by appearance-related insecurity (Zhu et al., 2021), thereby increasing their susceptibility to engage in ruminative thinking characterized by persistent and repetitive negative patterns (Neff, 2009). Therefore, we hypothesize that facial negative physical self may also contribute to increased rumination. Previous research has indicated that individuals with elevated levels of rumination tend to dwell excessively on negative social interactions and engage in harsh self-criticism regarding their behavioral performance, which exacerbates anxiety in interpersonal contexts and leads to social anxiety (Modini and Abbott, 2017). Furthermore, numerous empirical studies have consistently demonstrated a significant positive correlation between rumination and social anxiety (Brozovich et al., 2015; Abdollahi, 2019; Elhai et al., 2018; Valena et al., 2015; Edgar et al., 2024). Based on this evidence, the present study proposes Hypothesis 2: Rumination mediates the relationship between facial negative physical self and social anxiety (H2).

### 1.4 The regulating role of self-compassion

Self-compassion refers to an individual’s positive and caring attitude toward themselves when confronted with failures and personal shortcomings. According to Neff (2003), self-compassion stems from the cultivation of mindfulness, self-kindness, and a sense of common humanity. Consequently, individuals with high self-compassion are more likely to accept their imperfections and approach challenges by neither avoiding nor over-identifying with their issues, thereby mitigating ruminative thinking (Wasylkiw et al., 2012). Studies suggest that enhancing self-compassion serves as a potential protective strategy for populations prone to ruminative cognitive patterns. Specifically, self-compassion training or targeted interventions can effectively reduce individuals’ tendencies toward rumination (Neff, 2009). Therefore, individuals with higher levels of self-compassion can reduce their rumination in social situations, reducing their self-attention and reducing the frequency of self-criticism (Neff, 2009; Blackie and Kocovski, 2018). Moreover, social anxiety leads individuals to overestimate the likelihood that others are constantly noticing and negatively evaluating them (Werner et al., 2012), significantly impairing their ability to cope with everyday social situations. However, research has found that individuals with higher levels of self-compassion demonstrate greater insight into negative circumstances, exhibit enhanced self-acceptance, and maintain a kind and understanding attitude toward themselves, thereby mitigating social anxiety (Fink-Lamotte et al., 2023). Therefore, we speculate that in the relationship between rumination and social anxiety, self-compassion, as a positive emotion regulation strategy, can effectively alleviate the negative effects of rumination on social anxiety. Based on this, this study proposed the hypothesis that self-compassion plays a negative moderating role between rumination and social anxiety (H3).

**Figure 1:**
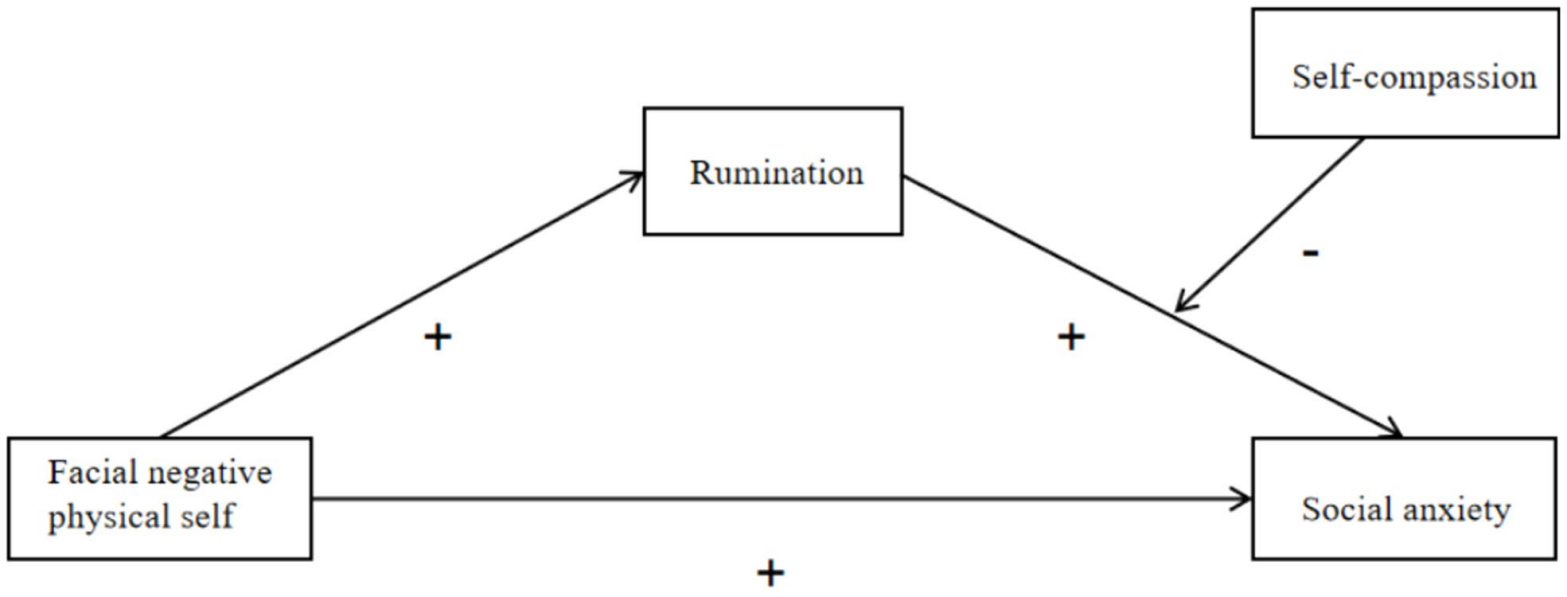
Hypothetical model diagram.

The specific hypothetical model is shown in Figure 1.

## 2 Materials and methods

### 2.1 Participants and procedure

This cross-sectional survey was conducted in Southwest China during October 2022. Participants were selected from a multi-disciplinary student population using a convenience sampling approach. The adoption of this non-probability sampling strategy aligns with Etikan et al.'s (2016) methodological framework, whereby convenience sampling is justified when the target population inherently meets predetermined eligibility criteria. All sampled students satisfied the study’s inclusion requirements prior to questionnaire administration. Inclusion criteria: college students at school; signed an informed consent form and voluntarily joined the study. Exclusion criteria: those with severe mental disorders that prevented them from cooperating with the survey; those who did not wish to join the study. The researcher distributed the questionnaire star QR code or link to the subjects through social media platforms, and the participants voluntarily filled in the questionnaire after reading the informed consent form. This study received approval from the North Sichuan Medical College. This study was not preregistered. As such, the confirmatory analyses should be interpreted with caution. A total of 1,350 questionnaires were sent out, the recovery rate was 95.3%, and the completion time was controlled within 15–20 min. After screening (there are 4 lie detector questions, and the wrong answers are regarded as invalid papers), there are 1,178 effective questionnaires, with an effective rate of 91.7%. The subjects were mainly from colleges and universities in Sichuan Province, including 492 male students (41.7%) and 686 female students (58.2%). There are 130 freshmen (11.0%), 347 sophomores (29.4%), 372 juniors (31.6%), 180 seniors (15.3%) and 149 graduate students (12.6%). 508 (43. 1%) of the students came from rural areas and 670 (56.8%) from urban areas.

### 2.2 Materials

#### 2.2.1 Facial negative physical self

The study utilized the Negative Physical Self Scale-Facial Appearance Subscale (NPSS-A; Chen, 2006). There are 11 questions with 5-point scoring. Higher scores indicate a higher degree of negative appearance. The Cronbach's α coefficient for this subscale in the current study was 0.92.

#### 2.2.2 Social anxiety

The level of social anxiety was assessed using the Interaction Anxiousness Scale (IAS; Leary, 1983) via self-report. The scale was developed by Leary using the clinical empirical method to rate an individual's level of social anxiety and consists of 15 questions on a 5-point scale, including 4 reverse scoring questions (3, 6, 10, and 15). Chunzi Peng revised it in 2004, and the revised scale has good reliability and validity and is a valid tool for measuring social anxiety in college students. The Cronbach's α coefficient for the scale in the current study was 0.85.

#### 2.2.3 Self-compassion

The study used the Adolescent Self-Compassion Scale (SCS) developed by Gong et al. (2014). Using the scale developed by Neff (2003) as a blueprint, Gong revamped the Adolescent Self-Compassion Scale, taking into account the cultural differences between China and the rest of the world. The scale consists of 12 items with the same degree of content encompassment as the scale compiled by Neff, i.e., self-kindness, common humanity, and mindfulness. 5-point scoring with 5 reverse scoring questions (2, 3, 4, 8, and 11), and the Cronbach's α coefficient for this scale in the current study was 0.66.

#### 2.2.4 Rumination

The Chinese version of the Ruminative Response Scale (RRS) developed by Nolen-Hoeksema (1991) and translated and revised by Han and Yang (2009) was used. There are 22 questions in total, and the scale is divided into four levels of scores from 1 to 4, with 1 representing "never" and 4 representing "always", and the higher the score, the higher the degree of rumination. The Cronbach's α coefficient for this scale in the current study was 0.93.

### 2.3 Analysis methods

SPSS 26.0 was used for statistical processing of data in this study. Relationships among the four variables and differences in demographic variables were explored. Next, the hypothesized model constructed in this study involves three tests of direct effect, mediating effect, and moderating effect, using the PROCESS plug-in to explore the mediating role of rumination and the moderating role of self-compassion. The PROCESS macro was employed for mediation analysis in preference to structural equation modeling (SEM) based on two methodological rationales. First, this computational tool offers enhanced operational efficiency through its pre-specified mediation models integrated with bootstrapping procedures, which has gained widespread recognition in contemporary mediation research (Preacher and Hayes, 2008). Second, empirical evidence from Hayes et al. (2017) confirms analytical consistency between PROCESS-derived results and SEM outputs in testing mediation pathways.

## 3 Results

### 3.1 Common method bias test

Four variables were measured in this study using questionnaires. Harman's one-factor method was used for testing. The results showed that nine factors with eigenroots greater than 1 were extracted from this study without variance rotation of the variables, and the variance explained by the first factor was 27% (< 40%). So there is no serious common method bias.

### 3.2 Correlation analyses

The correlations of the four variables were analyzed using Pearson correlation analysis and the results are shown in Table 1. As can be seen in Table 1, self-compassion and each factor were significantly correlated two-by-two with the other three variables. Specifically, self-compassion was negatively correlated with rumination (*p* < 0.01), facial negative physical self (*p* < 0.01), and social anxiety (*p* < 0.01), with correlation coefficients of –0.42, –0.38, and –0.39, respectively. Facial negative physical self was positively correlated with rumination (*p* < 0.01), with a coefficient of 0.57. Facial negative physical self and social anxiety were positively correlated (*p* < 0.01) with a coefficient of 0.44. Rumination thinking was positively correlated (*p* < 0.01) with social anxiety with a coefficient of 0.52.

**Table 1:** Correlation analysis for the variables include in the study.

Variable	1	2	3	4	5	6	7
1. Self-compassion	1						
2. Mindfullness	0.823^**^	1					
3. Self-kindness	0.77^**^	0.54^**^	1				
4. Common humanity	0.68^**^	0.25^**^	0.29^**^	1			
5. Rumination	-0.42^**^	−0.26^**^	−0.24^**^	−0.45^**^	1		
6. Facial negative physical self	−0.38^**^	−0.25^**^	−0.23^**^	−0.36^**^	0.57^**^	1	
7. Social anxiety	−0.39^**^	−0.20^**^	−0.28^**^	−0.43^**^	0.52^**^	0.44^**^	1

### 3.3 Mediating effects analysis

The hypothesized model constructed in this study involves three tests of direct effect, mediating effect, and moderating effect, and SPSS 26.0 was used to analyze the data to verify the previous hypotheses. To test the hypotheses proposed by the study, we proposed to use Process in SPSS for model testing. We controlled for gender and grade as demographic variables, and we verified the significance of the mediating effect of rumination in the facial negative physical self and social anxiety by using facial negative physical self as the independent variable and social anxiety as the dependent variable.The results showed (as shown in Table 2) that when we tested with Model 4 (simple mediator model), there was a significant positive predictive effect of facial negative physical self on social anxiety (*B* = 0.46, *t* = 17.66, *p* < 0.01), and Hypothesis H1 was supported: Facial negative physical self positively predicts social anxiety. We put rumination into the model as a mediating variable and the direct effect of the model remained significant (*B* = 0.38, *t* = 12.90, *p* < 0.01) and there was a significant positive predictive effect of facial negative physical self on rumination (*B* = 0.58, *t* = 24.08, *p* < 0.01), and rumination also positively predicted social anxiety (*B* = 0.24, *t* = 8.01, *p* < 0.01), suggesting that facial negative physical self enhances rumination and rumination enhances social anxiety. In addition, the direct utility of the facial negative physical self on social anxiety and the mediating utility of rumination were not included in the upper and lower bounds of the 95% confidence intervals in Bootstrap at zero (see Table 3). The results of this data suggest that the facial negative physical self not only directly predicts social anxiety, but also predicts social anxiety through the mediating effect of rumination, as validated by H2. Finally, the direct (0.236) and mediated (0.219) effects in the mediation model accounted for 51.9% and 48. 1% of the total effect (0.455), respectively. To test our hypotheses, we include moderating variables.

**Table 2:** Mediation modeling tests for rumination.

Regression (*N*=1178)		Fitness index			Significance	
Outcome variable	Predictor variables	*R*	*R* ^2^	*F*	*B*	*t*
Social anxiety		0.475	0.226	114.046**		
	Gender				0.332	6.318**
	Grade				0.026	1.423
	Facial negative physical self				0.455	17.658**
Rumination		0.576	0.332	194.542**		
	Gender				0.160	3.271**
	Grade				0.006	0.327**
	Facial negative physical self				0.576	24.083*
Social anxiety		0.567	0.322	139. 177**		
	Gender				0.271	5.492**
	Grade				0.024	1.397
	Rumination				0.236	8.013**
	Facial negative physical self				0.380	12.899**

*p < 0.05; **p < 0.01.

**Table 3:** Total effect, direct effect and indirect effect.

Effect	Effect size	BootSE	BootLLCI	BootULCI	Proportion (%)
Total effect	0.455	0.026	0.404	0.505	1
Direct effect	0.236	0.030	0.178	0.294	51.9%
Indirect effect	0.219	0.020	0.180	0.260	48.1%

### 3.4 Moderating effect analysis

**Figure 2:**
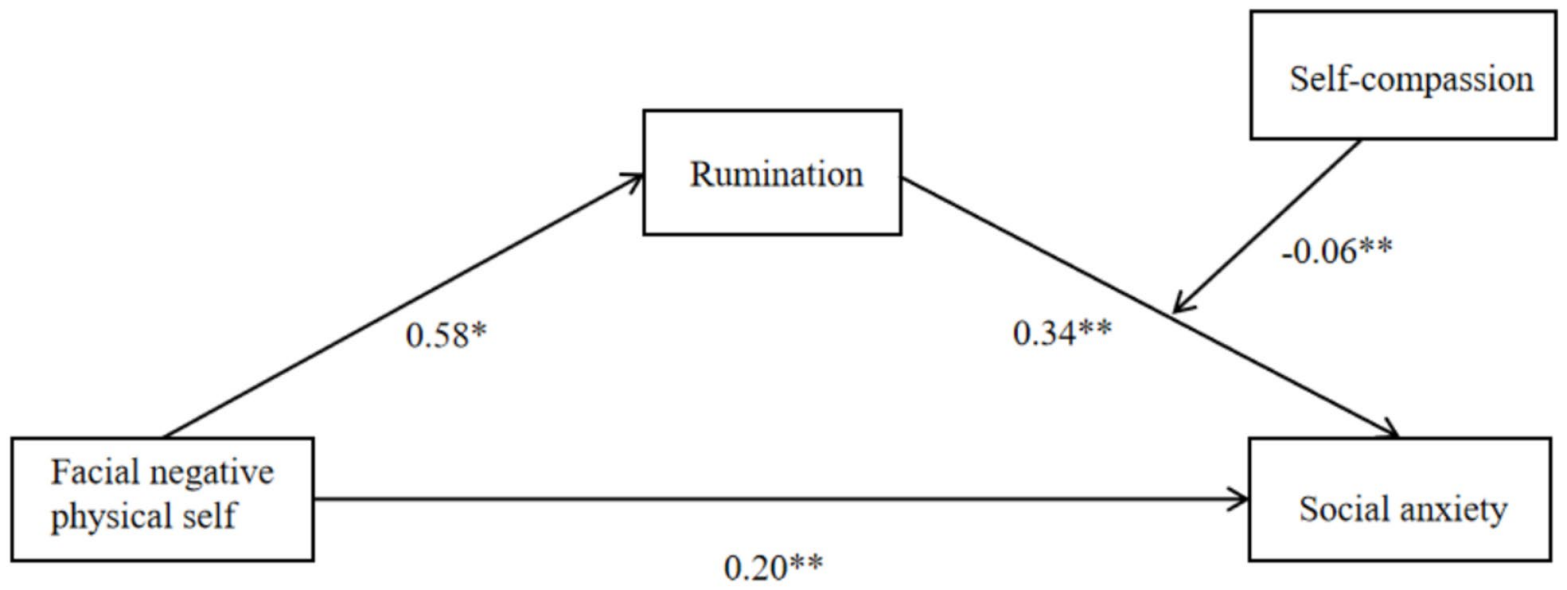
Moderated mediator model diagram. *p < 0.05; **p < 0.01.

We added self-compassion as a moderating variable to the path of the facial negative physical self effect of looks on social anxiety using Model 14 in the SPSS process (which assumes that the moderating variable moderates the second half of the path in a simple mediation model). After controlling for gender and grade, we tested this moderated mediation model.The results showed (see Table 4) that after placing self-compassion into the model, the interaction term between rumination and self-compassion had a significant negative effect on social anxiety (*β* = −0.06, *t* = 3.00, *p* < 0.01), and since rumination was a significant positive effect on social anxiety (*β* = 0.34, *t* = 11.19, *p* < 0.01), we concluded that self-compassion buffered the effect of rumination on the positive predictive effect of social anxiety. Combined with the simple slope test (see Figure 2), we found that college students with lower levels of self-compassion (one standard deviation below the mean, M-1SD), rumination was a significant positive predictor of social anxiety (simple slope = 0.28, *t* = 8.18, *p* < 0.01). In contrast, for individuals with higher levels of self-compassion (M + 1SD), rumination also significantly positively predicted social anxiety (simple slope = 0.39, *t* = 10.58, *p* < 0.01), but with a weaker predictive effect. This suggests a gradual weakening of the positive prediction of social anxiety by rumination due to increasing levels of individual self-compassion.

**Table 4:** Moderated mediation model test.

Regression (*N* = 1,178)		Fitness index			Significance	
Dependent variable	Independent variable	*R*	*R^2^*	*F*	*B*	*t*
Rumination		0.576	0.332	194.542**		
	Gender				0.160	3.271*
	Grade				0.006	0.327
	Facial negative physical self				0.576	24.083*
Social anxiety		0.592	0.351	105.387**		
	Gender				0.267	5.505**
	Grade				0.022	1.336
	Facial negative physical self				0.196	6.651**
	Rumination				0.336	11.194**
	Self-compassion				−0.167	−6.285**
	Rumination*Self-compassion				−0.058	2.996**

*p < 0.05; **p < 0.01.

**Figure A1:**
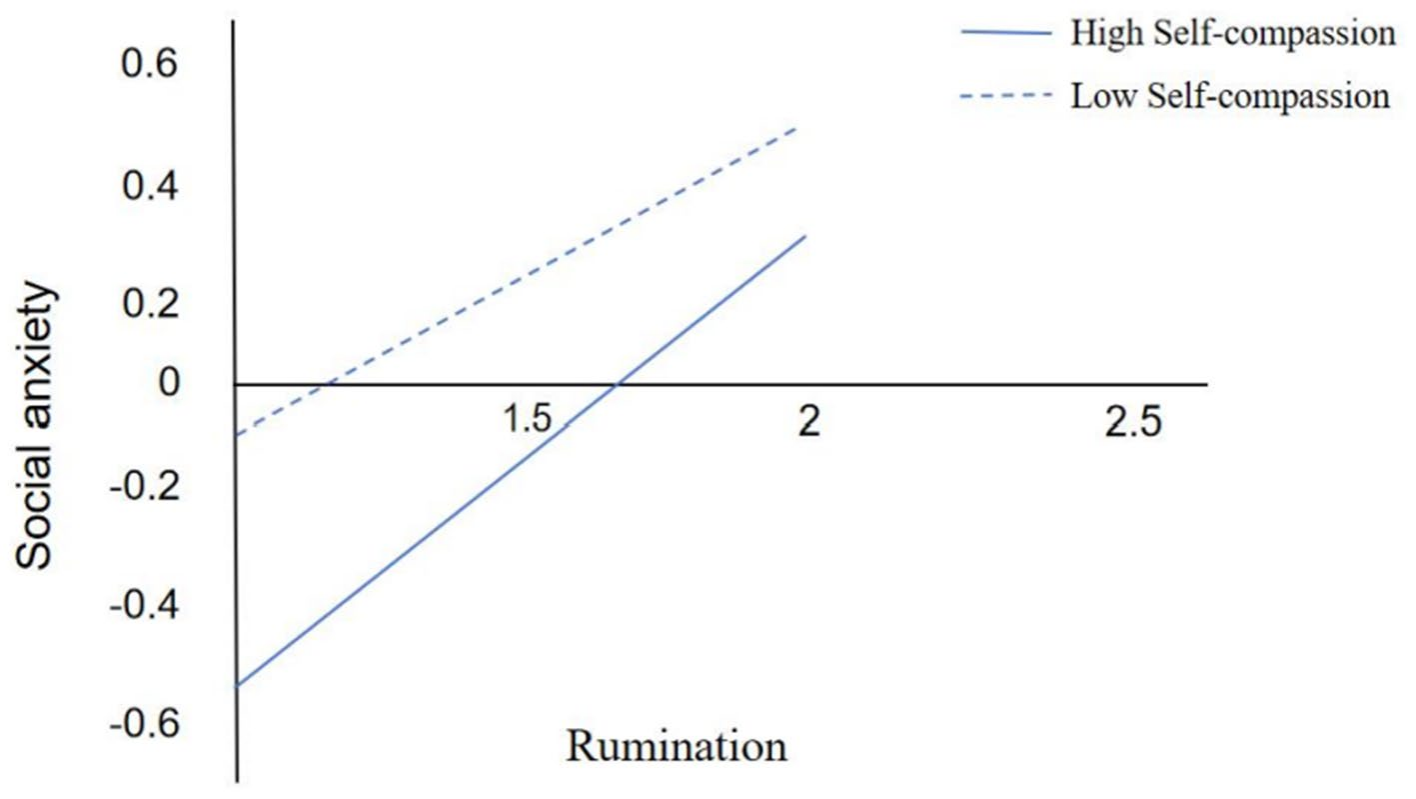
Interaction plot of moderating effects.

In addition, at all three levels of self-compassion, the mediating effect of rumination in the relationship between the look-negative body self and social anxiety tended to diminish as the level of self-compassion increased (see Table A1). This means that as college students’ level of self-compassion increased, the look-negative body self was less likely to influence social anxiety through rumination. This suggests that self-compassion moderated the second half of the mediating effect: self-compassion moderated the effect of rumination on social anxiety, as validated by H3. The specific moderated mediation model diagram is shown in Figure A1.

**Table A1:** Direct and mediating effects at different levels of self-compassion.

Effect	Level	Effect size	BootSE	BootLLCI	BootULCI
Direct effect		0.20	0.03	0.14	0.25
	M-1SD	0.160	0.022	0.118	0.203
Indirect effect	M	0.194	0.020	0.156	0.233
	M+1SD	0.227	0.022	0.186	0.271

## 4 Discussion

The present study found that facial negative physical self positively predicts social anxiety among college students, with rumination serving as a mediating factor in this relationship. Specifically, the influence of facial negative physical self on social anxiety may be realized through rumination. Self-compassion was found to moderate the latter half of this mediating pathway, that is, self-compassion moderates the effect of rumination on social anxiety. These findings provide empirical evidence for the prevention and intervention of social anxiety in college students.

### 4.1 The effect of facial negative physical self on social anxiety

The results revealed a positive correlation between the two variables, with facial negative physical self significantly predicting social anxiety. Social anxiety encompasses specific fears arising from social situations, including fear of exposure to unfamiliar individuals, apprehension about being scrutinized by others, and feelings of shame (Swee et al., 2021). Consequently, individuals with negative self-perceptions of their appearance may overestimate the likelihood of receiving negative evaluations from others, thereby developing symptoms of social anxiety. The research results of this paper are consistent with many domestic and foreign research results. Zhang and Hu (2021) demonstrated that self-objectification exerts a significant effect on social anxiety through the mediating role of body dissatisfaction. Findings indicated that, except for the "thinness" dimension, other dimensions of negative physical self (global, weight, facial appearance, and height) were significantly positively correlated with social anxiety (Wang, 2017). International studies have consistently supported these conclusions. For instance, regression analyses revealed that women’s social anxiety is significantly negatively associated with body image (Ratnasari et al., 2021). Additional research has shown that both men and women dissatisfied with their physical appearance often experience negative emotions (e.g., anxiety and depression) and exhibit reluctance to engage in social relationships, thereby contributing to social anxiety (Zhu et al., 2021; Dou et al., 2023).

### 4.2 Analysis of the mediating role of rumination

The mediation model revealed that rumination plays a partial mediating role between facial negative physical self and social anxiety among college students: Facial negative physical self can directly influence social anxiety and also indirectly affect it through rumination. First, facial negative self reflects dissatisfaction with one’s appearance. When individuals are dissatisfied with their physical appearance, they are more likely to perceive their appearance as being scrutinized or even mocked by others in social situations (Menzel et al., 2010). This perception leads to heightened negative emotions, increasing reluctance to engage in social relationships (Zhu et al., 2021), ultimately fostering anxiety and fear toward social interactions and behaviors. This perspective has been widely supported by researchers (Zhu et al., 2021; Cai et al., 2024). Furthermore, facial negative physical self in college students can also impact social anxiety via rumination. Individuals with stronger negative self-perceptions of their appearance exhibit lower self-acceptance and greater susceptibility to negative emotional states (Zhu et al., 2021), as well as a tendency to ruminate excessively about their appearance during interpersonal interactions (Neff, 2009). Rumination is a common and critical issue faced by college students in social contexts. If individuals excessively focus on their negative emotions, thoughts, or behaviors during social interactions, repetitively and passively dwell on details and antecedents of social events, fixate on the causes and consequences of distressing social experiences, and struggle to disengage from these thoughts, they are more likely to experience anxiety in social situations (Modini and Abbott, 2017). Therefore, facial negative physical self among college students can also influence social anxiety through the mediating role of rumination.

### 4.3 Analysis of the moderating role of self-compassion

The moderated mediation model revealed that self-compassion significantly moderates the mediating role of rumination between facial negative physical self and social anxiety. Specifically, across three levels of self-compassion, the mediating effect of rumination in the relationship between facial negative physical self and social anxiety gradually diminished as self-compassion levels increased. In other words, college students with higher self-compassion are less likely to experience social anxiety through the pathway of facial negative physical self influencing rumination. On one hand, research indicates that self-compassion, as an adaptive emotion regulation strategy, can effectively alleviate ruminative thinking in individuals prone to ruminative cognitive patterns (Neff, 2009). On the other hand, from the perspective of the three dimensions of self-compassion (self-kindness, common humanity, and mindfulness), enhancing self-compassion enables individuals to approach both past and potential future events with self-kindness, recognition of shared human experiences, and mindful acceptance. To elaborate: Self-kindness refers to treating one’s shortcomings with benevolence rather than self-judgment. Common humanity involves acknowledging that imperfection, suffering, and failure are universal human experiences. Mindfulness entails observing problems objectively without avoidance or over-identification, maintaining a non-emotional and accepting stance. If individuals cultivate self-compassion, they are better able to observe their emotional states and personal flaws with objectivity and openness, thereby reducing rumination (Allen and Leary, 2010). These findings are corroborated by extensive empirical studies (Butz and Stahlberg, 2018; Neff, 2009; Allen and Knight, 2005). Consequently, higher levels of self-compassion lead to reduced rumination, and the subsequent decline in rumination alleviates fear of social interactions (Modini and Abbott, 2017). Thus, self-compassion moderates the role of rumination in the association between facial negative physical self and social anxiety among college students.

## 5 Limitations and future directions

This study has several limitations. First, during data collection, the majority of participants were first- and second-year undergraduates, resulting in an uneven distribution across academic years. Second, as a cross-sectional study, it cannot establish causal relationships or fully demonstrate the predictive effect of negative facial appearance self-perception on college students' social anxiety. Third, regarding the study sample: (1) focusing solely on enrolled college students limits the sample representativeness, and the findings may not be generalizable to other populations; (2) participants were recruited exclusively from universities in Sichuan, China, which may introduce demographic limitations due to the single data source. Lastly, the lack of preregistration raises the possibility of file-drawer effects, limiting the robustness and replicability of our conclusions.

The limitations of this study provide concrete directions for future research. First, subsequent studies should strictly control the number of participants from each academic year to achieve balanced distribution across grade levels, thereby enhancing sample representativeness and result reliability. Second, building on our findings, future research could employ longitudinal intervention designs to examine how state self-compassion levels moderate the relationship between facial negative physical self and social anxiety. Third, expanding both the participant pool and geographical coverage would improve the generalizability of findings. Fourth, as negative physical self encompasses five dimensions (facial appearance, height, weight, thinness, and general negative physical self), future studies could focus on dimensions beyond facial appearance to investigate their differential impacts on social anxiety. Lastly, it is essential to preregister all procedures and hypotheses. This will be crucial in examining whether the results obtained in this study are robust and can be replicated.

## 6 Conclusion

This study revealed that: the facial negative physical self positively predicts social anxiety among college students; the facial negative physical self indirectly influences social anxiety through the mediating role of rumination; Self-compassion moderates the impact of rumination on social anxiety. These findings suggest that universities should pay attention to the phenomenon of appearance anxiety among college students, guiding them to establish appropriate self-evaluation standards. Rather than judging self-worth solely by physical appearance, students should be encouraged to conduct comprehensive assessments of their abilities and intrinsic values to cultivate inner self-confidence. Simultaneously, social media and popular culture should take responsibility for actively promoting healthy and diverse aesthetic standards to alleviate adolescents' appearance anxiety. Furthermore, this study provides intervention strategies for reducing college students' social anxiety, specifically through the enhancement of self-compassion competencies to optimize psychological well-being in social interactions.

## Data Availability

The raw data supporting the conclusions of this article will be made available by the authors, without undue reservation.
